# Clinical Characteristics, Prognostic Factors, and Thrombotic and Bleeding Outcomes in Philadelphia Chromosome-Negative Myeloproliferative Neoplasms: A Single-Center Cohort Study in Thailand

**DOI:** 10.7759/cureus.82141

**Published:** 2025-04-12

**Authors:** Suprang Suttantapidok, Weerapat Owattanapanich

**Affiliations:** 1 Department of Medicine, Vachira Phuket Hospital, Phuket, THA; 2 Department of Medicine, Division of Hematology, Faculty of Medicine Siriraj Hospital, Mahidol University, Bangkok, THA

**Keywords:** jak2v617f mutation, major bleeding, ph-mpns, prognostic models, thrombosis

## Abstract

Background

Patients with Philadelphia chromosome-negative myeloproliferative neoplasms (Ph-neg MPNs), including polycythemia vera (PV), essential thrombocythemia (ET), and primary myelofibrosis (PMF), face elevated risks of thrombosis and major bleeding. Because of these potentially severe complications, antiplatelet and anticoagulant therapies are often employed. This study aimed to evaluate thrombotic and bleeding events, identify associated risk factors, assess prognostic risk models, and investigate the effects of antithrombotic therapy in a Thai cohort of patients with Ph-neg MPNs.

Methods

This single-center cohort study in Thailand enrolled patients with Ph-neg MPNs from 2013 to 2023. Clinical characteristics, prognostic risk models (International Prognostic Scoring System (IPSS), International Prognostic Score for Essential Thrombocythemia (IPSET)-thrombosis, and Dynamic International Prognostic Scoring System (DIPSS)), and arterial and venous thrombotic events and bleeding complications were analyzed using descriptive statistics, logistic regression, and survival analysis.

Results

Among 173 patients, PV was the most common subtype (n = 111, 64.2%), with a male predominance and a median age of 57 years. Thrombotic events occurred in 36.9% (n = 41) of PV, 21.4% (n = 3) of PMF, and 18.8% (n = 9) of ET cases, with arterial thrombosis, particularly ischemic stroke, being the most frequent. The *JAK2^V617F^* mutation was prevalent in 72.5% (n = 79) of PV, 70.8% (n = 34) of ET, and 50% (n = 5) of PMF patients. Notably, no major bleeding events were observed despite intensive antithrombotic therapy. Multivariable analysis revealed that prior ischemic stroke (OR 22.51, *P *= 0.007) and dizziness/headache (OR 7.26, *P* = 0.022) were significant risk factors for thrombosis. Overall survival (OS) varied by disease subtype. PV patients had a five-year OS of 94.9% and a 10-year OS of 87.2%. ET patients demonstrated a five-year OS of 77.4%. PMF patients had the lowest survival, with a five-year OS of 67.7%.

Conclusions

This cohort study offers important insights into the clinical characteristics and complication profiles of Ph-neg MPNs in a Thai population. Compared with Western cohorts, patients in this study exhibited a higher incidence of thrombotic events, particularly ischemic stroke. These findings emphasize the need to consider regional variations in disease presentation and underscore the value of individualized risk stratification to optimize patient management in diverse clinical settings.

## Introduction

Philadelphia chromosome-negative myeloproliferative neoplasms (Ph-neg MPNs) are a diverse group of rare clonal disorders arising from hematopoietic stem cells. According to the 2016 World Health Organization (WHO) classification, Ph-neg MPNs include essential thrombocythemia (ET), polycythemia vera (PV), and primary myelofibrosis (PMF) [1]. Less common entities, such as chronic neutrophilic leukemia, hypereosinophilic syndrome, mastocytosis, and unclassifiable MPNs, also fall within this category. In the new edition of the 2022 WHO classification, Ph-neg MPNs include ET, PV, and PMF. Less common entities, such as chronic neutrophilic leukemia, chronic eosinophilic leukemia, juvenile myelomonocytic leukemia, and myeloproliferative neoplasm, not otherwise specified, also fall within this category [2].

The *JAK2^V617F^* mutation is present in approximately 95% of patients with PV and in 50%‒60% of those with ET or PMF [3]. Somatic mutations in *JAK2* exon 12 are identified in a subset of patients with PV [4,5]. Consequently, nearly all individuals with PV exhibit somatic *JAK2* mutations, whereas about half of those with ET or PMF do not harbor *JAK2* or *MPL* (myeloproliferative leukemia) alterations [6,7].

Patients with Ph-neg MPNs carry a significantly elevated risk of thrombotic and thromboembolic events compared to the general population, which substantially contributes to morbidity and mortality [8-13]. Conversely, these patients also show an increased risk of bleeding complications. Acquired von Willebrand syndrome, caused by excessive thrombocytosis, is a key driver of disease-related bleeding [14-17]. The significance of regulating the WBC count has been highlighted [18]. The recently published European Leukemia Net 2021 guideline recommends the consideration of cytoreductive therapy in patients with low-risk PV who have a persistent WBC count above 20.0 × 10^9^/L. Furthermore, the guidelines recommend considering cytoreductive therapy for those with WBC counts above 15.0 × 10^9^/L [19], indicating a shift in policy toward the treatment of high WBC counts. The results of the present study indicate that WBC control may be useful in the prevention of hemorrhagic events. This finding supports a previous study that reported a correlation between the *JAK2^V617F^* allele burden and the WBC count at the time of PV diagnosis [20]. Moreover, therapy-related factors, particularly the use of antiplatelet and anticoagulant agents in high-risk patients, further influence bleeding risk. As a result, bleeding risk assessments in MPNs must consider both disease-specific and treatment-related factors.

The pathogenesis of thromboembolic events in patients with Ph-neg MPNs is complex and has become increasingly elucidated over the past decade. Clinical factors such as advanced age, prior thrombotic events, obesity, hypertension, and hyperlipidemia, along with elevated blood cell counts (e.g., leukocytosis, erythrocytosis, and thrombocytosis), contribute to the heightened risk of thrombosis [21]. The *JAK2^V617F^* mutation is a notable risk factor for thrombotic events, especially compared with *JAK2*-negative cases [22]. In contrast, *MPL* and calreticulin (*CALR*) mutations are not linked to increased thrombotic risk. Research shows that *CALR*-mutated patients, despite often presenting with higher platelet counts, have a lower risk of thrombosis than *JAK2*-mutated patients [23]. Similarly, while *MPL* mutations appear in some MPN cases, studies suggest they do not confer an elevated thrombotic risk [24].

In current practice, patients with PV or ET are categorized as high risk or low risk based on age and previous thrombotic events [25]. Those over 60 years of age or with a prior thrombotic history are classified as high risk. The International Prognostic Score for ET-Thrombosis (IPSET-thrombosis) provides further risk stratification for ET [26]. Meanwhile, in PMF, risk assessment begins with the International Prognostic Scoring System (IPSS) for newly diagnosed patients and the Dynamic IPSS (DIPSS) for those in later disease stages. This serves as an independent risk factor for survival in patients with PMF. These models are enhanced by evaluating cytogenetic findings and transfusion status [27-30].

In a previous meta-analysis of 29 cohort studies involving 13,436 patients with MPN, the pooled prevalence of overall arterial and venous thrombosis was 16.2% and 6.2%, respectively, while the pooled prevalence of hemorrhagic complications was 6.2%. These findings indicate that thrombosis and bleeding are common initial manifestations of MPN [31]. However, data from Thailand remain scarce, and genetic characteristics may influence the risk of thrombosis and bleeding. Therefore, this study aimed to describe the clinical features and incidence of thrombotic and major bleeding events occurring before or during diagnosis in patients with Ph-neg MPNs, called the primary outcome, and to identify potential risk factors contributing to these events. The analysis emphasized underlying diagnoses, treatment modalities, and patient-related factors that may affect the occurrence of vascular events in Ph-neg MPN, which were considered the secondary outcome.

## Materials and methods

Study design and setting

We conducted a single-center study of patients with Ph-neg MPNs at Vachira Phuket Hospital, a community hospital in Thailand.

Ethical considerations

The study protocol, including the establishment of a Ph-neg MPN registry, was approved by the Research Ethics Committee of Vachira Phuket Hospital (approval no.: 023B2023). All study procedures followed the principles of the Declaration of Helsinki.

Study population

Patients were eligible for inclusion if they were at least 18 years old and had a confirmed diagnosis of a Ph-neg MPN, specifically PV, ET, or PMF (including pre-fibrotic and overt forms), based on the WHO classification (2008 or 2016) or the International Working Group for Myelofibrosis Research and Treatment criteria. Data were collected from January 2013 to August 2023.

Data collection

Baseline demographic and clinical data were obtained, including presenting signs and symptoms, laboratory findings, molecular genetic profiles, and complications. Data on concomitant medications, Ph-neg MPN-specific treatments, and thrombotic and major bleeding events (occurring before or during diagnosis) were also recorded.

Risk stratification models

Risk stratification was performed using disease-specific prognostic models. For PV, the IPSS was applied [32]. The IPSET-thrombosis model was used for ET to assess thrombotic risk [26]. For PMF, the risk was assessed using the IPSS at diagnosis and the DIPSS during disease progression to estimate overall survival (OS) [27-30].

Definitions of outcomes

Arterial thrombotic events were defined as acute ischemic stroke, acute myocardial infarction, peripheral arterial disease, acute limb ischemia, transient ischemic attack, or thrombosis at unusual sites (e.g., intra-abdominal thrombosis). Venous thrombosis included deep vein thrombosis, pulmonary embolism, or thrombosis at unusual sites such as cerebral venous sinuses and the splenic vein. Thrombotic events were diagnosed based on clinical presentation and imaging studies. Major bleeding events were defined as intracranial or retroperitoneal hemorrhages or bleeding episodes associated with a hemoglobin decrease of ≥ 2 g/dL or requiring transfusions [33].

Sample size calculation

We calculated the sample size based on the estimated incidence of thromboembolism. A previous study reported an incidence of 17.8% [34]. Using a 95% confidence level and a 6.5% allowable error, we determined a minimum requirement of 134 subjects. Allowing for 5% incomplete data, we targeted an overall sample size of 142.

Statistical analysis

Clinical data were analyzed using IBM SPSS Statistics for Windows, Version 29 (Released 2023; IBM Corp., Armonk, New York). Descriptive statistics are presented as median (range) for continuous variables and n (%) for categorical variables. The chi-square test and Kruskal‒Wallis test were used to compare categorical and continuous data across MPN subtypes, respectively. We employed multiple logistic regression to identify predictors of thrombotic events, including all predefined variables with *P* < 0.01 on univariable analysis. OS was estimated using the Kaplan‒Meier method. Two-sided *P-*values < 0.05 were considered statistically significant in all tests.

## Results

Cohort composition

A total of 173 patients with Ph-neg MPNs were analyzed. Of these, 111 had PV (64.2%), 48 had ET (27.7%), and 14 had PMF (8.1%). The median ages were 57 years for PV, 66.5 years for ET, and 72 years for PMF. PV was more common in males.

Clinical presentations

Asymptomatic presentations were observed in 48.6% of PV cases, 77.1% of ET cases, and 42.9% of PMF cases. Among symptomatic PV patients, dizziness or headache (18.9%), hemiparesis (18.0%), and splenomegaly (3.6%) were predominant. ET patients also reported dizziness or headache (10.4%), hemiparesis (8.3%), and splenomegaly (4.2%). In contrast, PMF patients most frequently presented with splenomegaly (35.7%), fatigue (28.6%), dizziness (14.3%), and abdominal symptoms (14.3%). Baseline characteristics are summarized in Table 1, along with the hematologic parameters.

**Table 1:** Baseline characteristics of patients with Philadelphia chromosome-negative myeloproliferative neoplasms Data are reported as median (range) for continuous variables and n (%) for categorical variables. Comparisons were conducted using the chi-square test for categorical variables and the Kruskal-Wallis test for continuous variables. WBC, white blood cell count

Variables	All Patients	Polycythemia Vera	Essential Thrombocythemia	Primary Myelofibrosis
Number (%)	173 (100%)	111 (64.2%)	48 (27.7%)	14 (8.1%)
Age (years), median (range)	61 (25, 96)	57 (25, 87)	66.5 (30, 96)	72 (40, 80)
Male sex	111 (64.2%)	81 (73.0%)	24 (50.0%)	6 (42.9%)
Symptoms, n (%)				
Asymptomatic (checkup-found)	32 (18.5%)	15 (13.5%)	14 (29.2%)	3 (21.4%)
Dizziness/headache	28 (16.2%)	21 (18.9%)	5 (10.4%)	2 (14.3%)
Hemiparesis	25 (14.5%)	20 (18.0%)	4 (8.3%)	1 (7.1%)
Splenomegaly	11 (6.4%)	4 (3.6%)	2 (4.2%)	5 (35.7%)
Fatigue	8 (4.6%)	3 (2.7%)	1 (2.1%)	4 (28.6%)
Abdominal symptoms	6 (3.5%)	4 (3.6%)	0 (0%)	2 (14.3%)
Erythromelalgia	4 (2.3%)	3 (2.7%)	1 (2.1%)	0 (0%)
Numbness	3 (1.7%)	2 (1.8%)	0 (0%)	1 (7.1%)
Comorbidity, n (%)				
Hypertension	94 (54.3%)	65 (58.6%)	22 (45.8%)	7 (50.0%)
Dyslipidemia	52 (30.1%)	28 (25.2%)	19 (39.6%)	5 (35.7%)
Diabetes mellitus	29 (16.8%)	17 (15.3%)	10 (20.8%)	2 (14.3%)
Cardiovascular disease	14 (8.1%)	10 (9.0%)	2 (4.2%)	2 (14.3%)
Previous ischemic stroke	14 (8.1%)	10 (9.0%)	3 (6.3%)	1 (7.1%)
Atrial fibrillation	3 (1.7%)	1 (0.9%)	1 (2.1%)	1 (7.1%)
Previous hemorrhagic stroke	2 (1.2%)	1 (0.9%)	1 (2.1%)	0 (0%)
Smoking	30 (17.3%)	28 (25.2%)	2 (4.2%)	0 (0%)
Laboratory (median, range)				
Hemoglobin (g/dL)	17.0 (4.1, 23.1)	18.9 (4.1, 23.1)	12.5 (7.3, 16.7)	9.9 (6.2, 16.3)
Hematocrit (%)	52.9 (13.2, 73.9)	58.8 (13.2, 73.9)	37.4 (24.3, 49.0)	30.1 (22.9, 52.3)
WBC count (×^10 ^U/L)	12.5 (3.3, 111.2)	11.8 (3.3, 111.2)	13.3 (4.4, 60.3)	21.7 (4.91, 61.5)
Platelet count (×^10^ U/L)	629 (73, 2511)	447 (80, 2511)	1069 (527, 2294)	577.5 (73.0, 1209.0)
​​​​​​​*JAK2^V617F^* mutation	n = 167	n = 109	n = 48	n = 10
Positive	118 (70.7%)	79 (72.5%)	34 (70.8%)	5 (50.0%)
Negative	49 (29.3%)	30 (27.5%)	14 (29.2%)	5 (50.0%)

Molecular findings

The *JAK2^V617F^* mutation was detected in a large proportion of patients across all subtypes, with prevalence rates of 72.5% in PV, 70.8% in ET, and 50% in PMF.

Thrombotic events

Table 2 summarizes the frequency of thrombotic events within our cohort. Approximately one-third of patients experienced arterial or venous thromboembolism, with arterial thrombosis occurring more frequently across all subtypes. The most common thrombotic events included acute ischemic stroke (64.2%), acute myocardial infarction (13.2%), transient ischemic attack (7.5%), and acute limb ischemia (5.7%).

**Table 2:** Thrombotic events before or at the time of diagnosis in patients with Philadelphia chromosome-negative myeloproliferative neoplasms Data are presented as n (%). Statistical comparisons were performed using the chi-square test.

Variables	All Patients (N = 173)	Polycythemia Vera (n = 111)	Essential Thrombocythemia (n = 48)	Primary Myelofibrosis (n = 14)
Thrombotic events pre/at diagnosis (yes)	53 (30.6%)	41 (36.9%)	9 (18.8%)	3 (21.4%)
Type of arterial thrombotic event pre/at diagnosis				
Acute ischemic stroke	34 (64.2%)	24 (58.5%)	8 (88.9%)	2 (66.7%)
Acute myocardial infarction	7 (13.2%)	6 (14.6%)	1 (11.1%)	0 (0)
Transient ischemic attack	4 (7.5%)	4 (9.8%)	0 (0)	0 (0)
Acute limb ischemia	3 (5.7%)	3 (7.3%)	0 (0)	0 (0)
Peripheral arterial disease	1 (1.9%)	0 (0)	0 (0)	1 (33.3%)
Arterial thrombosis at intraabdominal arteries	1 (1.9%)	1 (2.4%)	0 (0)	0 (0)
Type of venous thrombotic event pre/at diagnosis				
Cerebral venous sinus thrombosis	1 (1.9%)	1 (2.4%)	0 (0)	0 (0)
Splenic vein thrombosis/hepatic vein thrombosis	1 (1.9%)	1 (2.4%)	0 (0)	0 (0)
Deep vein thrombosis	1 (1.9%)	1 (2.4%)	0 (0)	0 (0)

Incidence by Ph-neg MPN subtype

PV patients had the highest prevalence of thrombotic events before or at diagnosis (36.9%), followed by those with PMF (21.4%) and ET (18.8%). Statistically significant differences were observed among subtypes. In PV, the predominant events were thrombotic events, which were acute ischemic stroke (58.5%), acute myocardial infarction (14.6%), and transient ischemic attack (9.8%). Among ET patients, nearly 90% of thrombotic episodes were acute ischemic strokes.

Venous thrombotic events before or at diagnosis were observed only in PV patients. Three cases were recorded: cerebral venous sinus thrombosis (2.4%), splenic or hepatic vein thrombosis (2.4%), and deep vein thrombosis (2.4%). No cases of pulmonary embolism were observed in the entire cohort. Additionally, no major bleeding events were recorded in the entire cohort.

Thrombotic and bleeding events in *JAK2^V617F^*-positive patients

Among patients harboring the *JAK2^V617F^* mutation, approximately one-third experienced thromboembolic events (Table 3). Thrombosis was most common in those with PMF (40%), followed by PV (36.7%) and ET (23.5%). No major bleeding events were reported in this subgroup.

**Table 3:** Thrombosis and bleeding events in JAK2V617F-positive patients with Philadelphia chromosome-negative myeloproliferative neoplasms Data are presented as n (%). Categorical variables were compared using the chi-square test. Low risk was defined as age < 60 years with no prior thrombotic events. High risk was defined as age ≥ 60 years and/or a history of thrombosis.

Factors	All Patients (N = 118)	Polycythemia Vera (n = 79)	Essential Thrombocythemia (n = 34)	Primary Myelofibrosis (n = 5)
Thrombotic event pre/at diagnosis (yes; n)	39 (33.1%)	29 (36.7%)	8 (23.5%)	2 (40.0%)
Acute ischemic stroke	24 (61.5%)	16 (55.2%)	7 (87.5%)	1 (50.0%)
Acute myocardial infarction	6 (15.4%)	5 (17.2%)	1 (12.5%)	0 (0%)
Peripheral arterial disease	1 (2.6%)	0 (0%)	0 (0%)	1 (50.0%)
Acute limb ischemia	3 (7.7%)	3 (10.3%)	0 (0%)	0 (0%)
Deep vein thrombosis	0 (0%)	0 (0%)	0 (0%)	0 (0%)
Cerebral venous sinus thrombosis	1 (2.6%)	1 (3.4%)	0 (0%)	0 (0%)
Splenic/hepatic vein thrombosis	1 (2.6%)	1 (3.4%)	0 (0%)	0 (0%)
Transient ischemic attack	3 (7.7%)	3 (10.3%)	0 (0%)	0 (0%)
Recurrent pregnancy loss	0 (0%)	0 (0%)	0 (0%)	0 (0%)
Arterial thrombosis at intra-abdominal arteries	0 (0%)	0 (0%)	0 (0%)	0 (0%)
Other	0 (0%)	0 (0%)	0 (0%)	0 (0%)
Bleeding event pre/at diagnosis (yes; n)	0 (0%)	0 (0%)	0 (0%)	0 (0%)
Cerebral hemorrhage	0 (0%)	0 (0%)	0 (0%)	0 (0%)
Gastrointestinal hemorrhage	0 (0%)	0 (0%)	0 (0%)	0 (0%)
Skin and/or mucosal bleeding	0 (0%)	0 (0%)	0 (0%)	0 (0%)
Other	-	-	-	-
Risk stratification (n = 113)				
Low risk	29 (25.7%)	19 (24.1%)	10 (29.4%)	-
High risk	84 (74.3%)	60 (75.9%)	24 (70.6%)	-

Prognostic risk stratification

**Figure 1:**
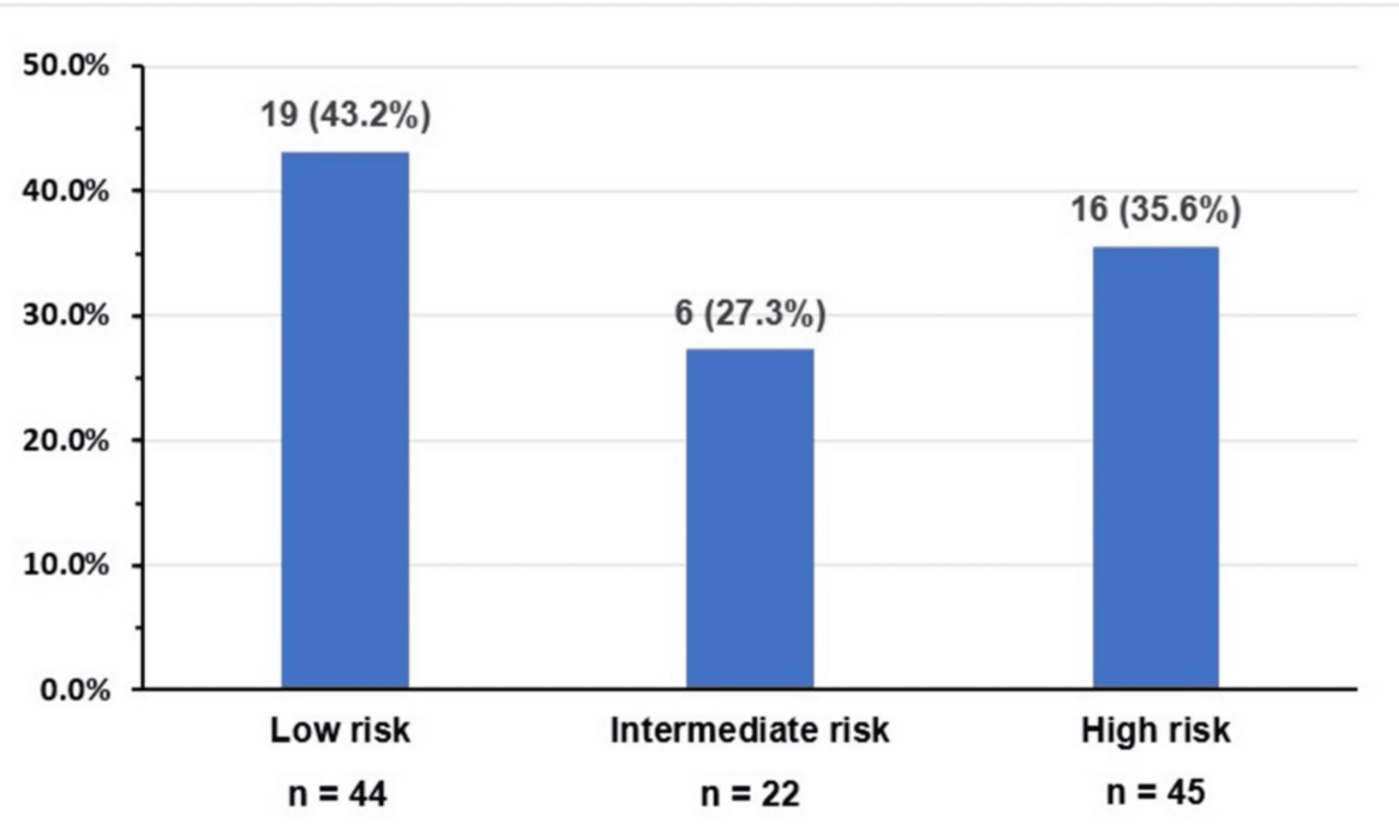
Thrombotic events in polycythemia vera patients according to International Prognostic Scoring System risk score (n = 111)

The distribution of patients across prognostic risk categories for PV, ET, and PMF is summarized below. In PV, risk stratification using the IPSS [32] showed that most patients were classified as either low risk (39.7%) or high risk (40.5%) (Table 4, Figure 1). The high-risk classification was primarily driven by older age and leukocytosis, despite the absence of prior venous thrombotic events in many patients.

**Table 4:** International Prognostic Scoring System risk stratification in patients with polycythemia vera Data are presented as n (%). IPSS, International Prognostic Scoring System; PV, polycythemia vera; WBC, white blood cell count

Variables	Polycythemia Vera (n = 111)
Age > 66 years (5 points; yes)	32 (28.8%)
Age 57‒66 years (2 points; yes)	24 (21.6%)
WBC > 15 x 10^9^/L (1 point; yes)	41 (36.9%)
Previous venous thrombosis (1 point; yes)	0 (0%)
IPSS score (PV)	
0 (low risk)	44 (39.7%)
I‒2 (intermediate risk)	22 (19.8%)
> 3 (high risk)	45 (40.5%)

**Figure 2:**
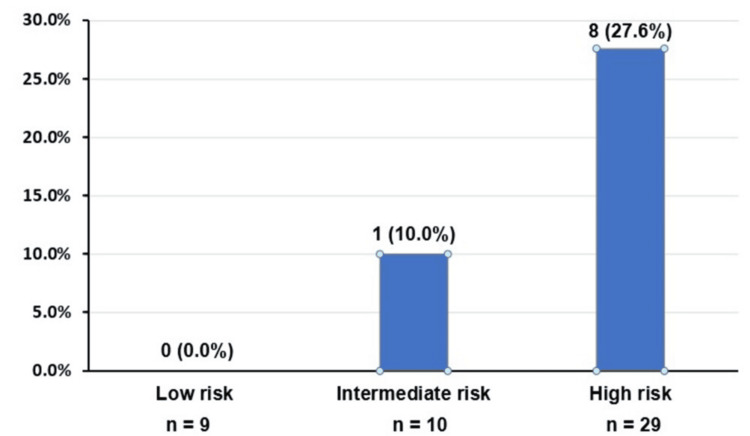
Thrombotic events in essential thrombocythemia patients according to International Prognostic Score for Essential Thrombocythemia–Thrombosis (n = 48)

Among ET patients, 60.4% were categorized as high risk according to the IPSET-thrombosis model (Table 5, Figure 2). This was largely due to a high prevalence of *JAK2^V617F^* mutations and older age, with many patients also having one or more cardiovascular risk factors.

**Table 5:** International Prognostic Score for Essential Thrombocythemia–Thrombosis in patients with essential thrombocythemia Data are presented as n (%). ET, essential thrombocythemia; IPSET-thrombosis, International Prognostic Score for Essential Thrombocythemia–Thrombosis

Variables	Essential Thrombocythemia (n = 48)
Age ≥ 60 years (1 point)	32 (66.7%)
Previous thrombotic event (2 points)	5 (10.4%)
Cardiovascular risk ≥ 1 (1 point)	30 (62.5%)
JAK2^V617F^ mutation (2 points)	34 (70.8%)
IPSET-thrombosis score	
0‒1 (low risk)	9 (18.8%)
2 (intermediate risk)	10 (20.8%)
≥ 3 (high risk)	29 (60.4%)

**Figure 3:**
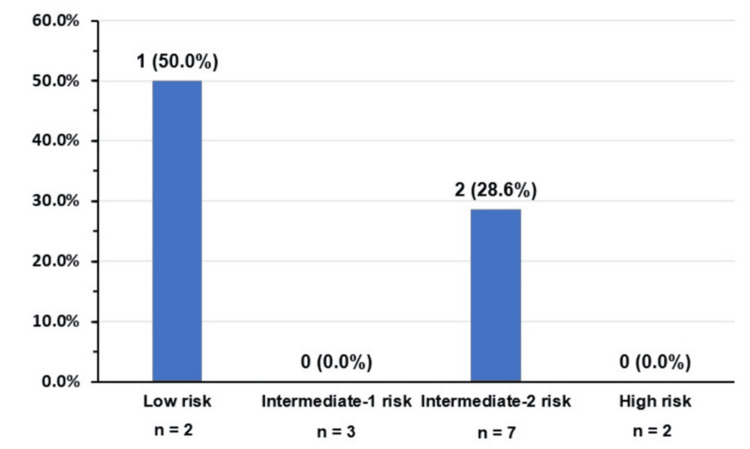
Thrombotic events in primary myelofibrosis patients according to Dynamic International Prognostic Scoring System risk score (n = 14)

**Figure 4:**
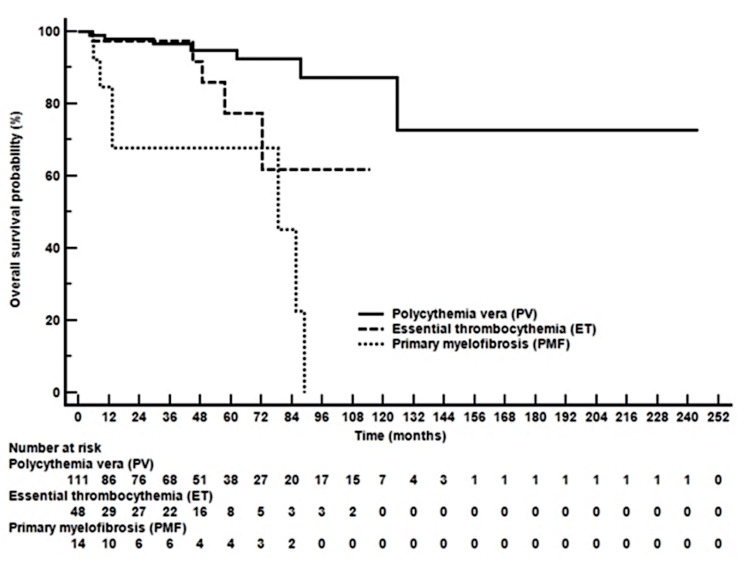
Overall survival of patients with Philadelphia chromosome-negative myeloproliferative neoplasms

For PMF, most patients were assigned to the intermediate-2 risk group using both the IPSS and the DIPSS (Table 6, Figure 3). These risk assignments reflected common clinical features such as anemia, leukocytosis, and constitutional symptoms. The use of DIPSS also enabled dynamic risk reassessment over the course of disease progression. The OS of patients with Ph-neg MPNs is shown in Figure 4.

**Table 6:** International Prognostic Scoring System and Dynamic International Prognostic Scoring System risk classification in patients with primary myelofibrosis Data are presented as n (%). DIPSS, Dynamic International Prognostic Scoring System; IPSS, International Prognostic Scoring System; PMF, primary myelofibrosis; WBC, white blood cell count

Variables	Primary Myelofibrosis (n = 14)
Age > 65 years	10 (71.4%)
Constitutional symptoms	7 (50.0%)
Hemoglobin < 10 g/dL	8 (57.1%)
WBC > 25 x 10^9^/L	6 (42.9%)
Circulating blasts ≥1%	0 (0%)
IPSS score	
0 (Low risk)	2 (14.3%)
1 (Intermediate-1 risk)	2 (14.3%)
2 (Intermediate-2 risk)	4 (28.6%)
≥ 3 (High risk)	6 (42.8%)
DIPSS (PMF) score	
0 (Low risk)	2 (14.3%)
1‒2 (Intermediate-1 risk)	3 (21.4%)
3‒4 (Intermediate-2 risk)	7 (50.0%)
5‒6 (High risk)	2 (14.3%)

Factors associated with thrombosis in PV and ET

Table 7 presents the factors associated with thrombosis in patients with PV and ET. In PV, dizziness or headache was strongly associated with thrombosis in both univariable analysis and multivariable analysis. Similarly, a history of ischemic stroke significantly increased the odds of thrombosis according to the univariable and multivariable analyses. No factors demonstrated a statistically significant association with thrombosis in the ET subgroup.

**Table 7:** Univariable and multivariable analyses of thrombosis risk factors in polycythemia vera and essential thrombocythemia *P < 0.05 is statistically significant. ET, essential thrombocythemia; OR, odds ratio; PV, polycythemia vera; CI: confidence interval

Factor	Univariable Analysis		Multivariable Analysis	
	Unadjusted OR (95% CI)	*P*-value	Adjusted OR (95% CI)	*P*-value
Risk factors of thrombosis in PV				
Dizziness/headache	7.265 (1.596, 33.07)	0.010*	7.262 (1.339, 39.377)	0.022*
Abdominal symptoms or splenomegaly	0.846 (0.148, 4.835)	0.851	-	-
Hemiparesis	N/A	< 0.001*	-	-
Cardiovascular disease	2.829 (0.748, 10.69)	0.168	-	-
Hypertension	1.624 (0.731, 3.609)	0.232	-	-
Diabetes mellitus	0.316 (0.085, 1.174)	0.102	-	-
Dyslipidemia	1.702 (0.713, 4.067)	0.229	-	-
Chronic kidney diseases	1.153 (0.305, 4.353)	1.000	-	-
Previous ischemic stroke	19.41 (2.357, 159.8)	<0.001*	22.51 (2.362, 214.43)	0.007*
Smoking	2.074 (0.868, 4.958)	0.098	1.630 (0.631, 4.209)	0.313
Leukocytosis (> 10.5 × 10^9^/L)	0.868 (0.392, 1.923)	0.727	-	-
Thrombocytosis (> 450 × 10^9^/L)	1.270 (0.586, 2.754)	0.545	-	-
Extreme thrombocytosis (> 1000 × 10^9^/L)	1.688 (0.622, 4.576)	0.301	-	-
JAK2^V617F^ mutation: positive	1.160 (0.478, 2.814)	0.743	-	-
Risk factors of thrombosis in ET				
Dizziness/headache	N/A	0.568	-	-
Abdominal symptoms or splenomegaly	4.750 (0.268, 84.175)	0.343	-	-
Hemiparesis	N/A	-	-	-
Cardiovascular disease	4.750 (0.268, 84.18)	0.343	-	-
Hypertension	0.933 (0.217, 4.010)	1.000	-	-
Diabetes mellitus	N/A	-	-	-
Dyslipidemia	1.280 (0.296, 5.537)	1.000	-	-
Chronic kidney diseases	N/A	-	-	-
Previous ischemic stroke	N/A	-	-	-
Smoking	4.750 (0.268, 84.18)	0.343	-	-
Leukocytosis (> 10.5 × 10^9^/L)	0.625 (0.143, 2.728)	0.701	-	-
Thrombocytosis (> 450 × 10^9^/L)	N/A	-	-	-
Extreme thrombocytosis (> 1000 × 10^9^/L)	0.870 (0.202, 3.750)	1.000	-	-
JAK2^V617F^ mutation: positive	4.000 (0.451, 35.49)	0.213	-	-

Antithrombotic and cytoreductive therapies

Antiplatelet and anticoagulant therapies were widely utilized in this cohort, with 99% of patients receiving acetylsalicylic acid (ASA). In terms of cytoreductive treatment, 49.2 % of patients were on hydroxyurea (Table 8).

**Table 8:** Antiplatelet, anticoagulant, and myeloproliferative neoplasm-specific therapies in patients with Philadelphia chromosome-negative myeloproliferative neoplasms Data are presented as n (%). Categorical variables were compared using the chi-square test.

Treatment	All Patients (N = 173)	Polycythemia Vera (n = 111)	Essential Thrombocythemia (n = 48)	Primary Myelofibrosis (n = 14)
Aspirin	169 (97.7%)	110 (99.1%)	48 (100%)	11 (78.6%)
Hydroxyurea	116 (67.1%)	74 (66.7%)	35 (72.9%)	7 (50.0%)

## Discussion

In our cohort, PV was the most common subtype (64.2%), followed by ET (27.7%) and PMF (8.1%), which was compatible with a report in Western populations [35]. We also observed a marked male predominance in PV (male-to-female ratio of 2.7:1). The median age at diagnosis in our series (PV: 57 years, ET: 66.5 years, PMF: 72 years) is younger than typically seen in Western patients (65-70 years). These findings suggest that environmental or genetic factors may favor an earlier onset of Ph-neg MPNs in Asian populations.

This study highlights a distinct pattern of clinical presentations and elevated thrombotic risks among Thai patients with Ph-neg MPNs. We observed thrombotic events in 36.9% of PV patients, 21% of PMF patients, and 18.8% of ET patients, with arterial thrombosis being the most common complication. These rates align with the meta-analysis by Rungjirajittranon et al. [31], which showed higher thrombotic risk in PV (28.6%) than in ET (20.7%) or PMF (9.5%). However, our cohort had a slightly elevated incidence of thrombotic events, suggesting possible regional differences in risk profiles or genetic predisposition.

Our findings indicate that ischemic stroke was the most prevalent arterial thrombotic event (64.2% of cases), aligning with the meta-analysis that also highlighted cerebrovascular events as a major complication in MPN. The presence of the *JAK2^V617F^* mutation was associated with an increased risk of thrombosis in our cohort (72.5% in PV, 70.8% in ET, and 50% in PMF), which corroborates prior studies [25,36], suggesting *JAK2^V617F^* as an independent risk factor for thrombosis. Similarly, a previous study [37] confirmed that patients with *JAK2* mutations face a significantly higher risk of thrombotic complications compared to non-mutated individuals.

The meta-analysis by Rungjirajittranon et al. [31] reported a thrombosis prevalence of 20.0%, including 16.2% arterial and 6.2% venous events. Cerebrovascular disease or transient ischemic attack, coronary heart disease, and deep venous thrombosis were most frequent. The analysis also noted hemorrhagic complications in 6.2% of patients, including gastrointestinal, mucosal, and cutaneous bleeding. In contrast, our study found a higher overall rate of thrombotic events, especially in PV patients (36.9%). The predominance of ischemic stroke in our cohort aligns with the meta-analysis, which also identified cerebrovascular events as the most common thrombotic complication in MPNs. However, we did not observe any major bleeding episodes, differing from the 6.2% bleeding rate reported in the meta-analysis. This discrepancy may reflect differences in patient selection, treatment strategies, or genetic factors affecting bleeding risk in our population.

Our findings also support previous evidence that leukocytosis contributes to thrombotic risk in MPNs. Our analysis shows 36.9% of PV patients had leukocytosis (> 10.5 × 10^9^/L). This factor has been linked to increased thrombosis in earlier studies [38]. Additionally, 60.4% of ET patients were classified as high-risk according to the IPSET-thrombosis model. This aligns with reports indicating that previous history of thrombosis, older age, and *JAK2* mutations together significantly heighten thrombotic risk [39].

Survival outcomes in our study are consistent with previous research. The five-year OS for PV (94.9%) and ET (77.4%) matched global rates [12]. By contrast, PMF had worse survival (67.7% at five years) due to its more aggressive nature. The meta-analysis similarly documented reduced survival in PMF compared with PV and ET, corroborating our observations.

Our analysis highlights the usefulness of combining disease-specific and cardiovascular predictors to achieve individualized risk stratification for improved MPN management. Future research should investigate genetic and epidemiological variations that influence thrombotic risk. Prognostic models should also be refined to enhance risk stratification and inform treatment decisions.

One key strength of this study lies in its comprehensive characterization of thrombotic events and associated risk factors within a single-center cohort. These findings offer valuable insights into MPN-related complications in a Southeast Asian population. The clearly delineated cohort with long-term follow-up enabled an in-depth evaluation of disease burden and prognostic factors. Furthermore, the use of validated prognostic models, such as IPSET-thrombosis and DIPSS, enhances the clinical applicability of our results. Our findings support individualized risk assessment and may help refine stratification strategies, guide therapeutic decision-making, and improve early identification and management, particularly in asymptomatic patients who present with erythrocytosis or thrombocytosis during routine health checkups.

Nonetheless, several limitations warrant consideration. First, the single-center, retrospective design may constrain the generalizability of the findings. Second, although the incidence of thrombotic events was high, the absence of major bleeding complications may reflect underestimation due to retrospective data collection. Third, other driver mutations, as well as non-driver mutations, were not comprehensively investigated, which may have affected risk stratification. Lastly, variations in treatment practices and patient selection criteria may contribute to differences between our findings and those reported in prior meta-analyses.

Despite these limitations, our study provides a valuable contribution to understanding the clinical spectrum of Ph-neg MPNs in a Southeast Asian context. Thrombosis emerged as a major complication, particularly in PV and PMF, with ischemic stroke being the most prevalent event. Multivariable analysis identified prior ischemic stroke and the presence of dizziness or headache as significant risk factors. These results highlight the importance of early recognition and individualized risk assessment, especially in PV patients presenting with subtle symptoms. The use of validated prognostic models, supported by long-term follow-up, reinforces the clinical applicability of these findings and may inform personalized management strategies, including early intervention for asymptomatic individuals identified during routine checkups.

## Conclusions

This cohort study offers important insights into the clinical characteristics and complication profiles of Ph-neg MPNs in a Thai population. Compared with Western cohorts, patients in this study exhibited a higher incidence of thrombotic events, particularly ischemic stroke. These findings emphasize the need to consider regional variations in disease presentation and underscore the value of individualized risk stratification to optimize patient management in diverse clinical settings.
